# 1090. Factors effecting mortality among Covid-19 patients in Renal Transplant Recipients from a single center in Pakistan

**DOI:** 10.1093/ofid/ofac492.930

**Published:** 2022-12-15

**Authors:** Asma Nasim, Sunil Kumar

**Affiliations:** Sindh Institute of Urology and Transplantation, Karachi, Sindh, Pakistan; Sindh Institute of Urology and Transplantation, Karachi, Sindh, Pakistan

## Abstract

**Background:**

Corona virus disease-19 (Covid-19) has significantly affected organ transplantation with concerns regarding severe infection and mortality. Data on Covid-19 in renal transplant recipients (RTRs) is scarce from Pakistan. The aim of this study is find out the factors effecting mortality among Covid-19 patients in renal transplant recipients from the largest transplant center of Pakistan.

**Methods:**

All RTRs >18 years, with positive severe acute respiratory syndrome coronavirus-2 (SARS-CoV-2) polymerase chain reaction (PCR) and diagnosed as severe disease, between April to December 2020 were retrospectively reviewed. The severe disease was defined as O2 saturation < 94% at room air on admission. Survivors and non- survivors were compared. Demographics, immunosuppression, comorbid conditions, clinical features, laboratory investigations and graft function were noted.

**Results:**

A total of 95 RTRs had severe disease. There was no difference in mortality between age, gender and co-morbid conditions among survivors and non-survivors. Both groups received similar immunosuppressive regimen. Intensive care unit (ICU) admission [16.5% vs 68.8% p< 0.001 OR 11.17 95% CI (3.3-37.6)] and high D-dimers >1.5µg/ml (p=0.052) at the time of admission were significantly associated with mortality. There was no association of graft function with mortality. Treatment with methyl-prednisolone was found to be significantly associated with survival [83% vs 43% P=0.02 OR 0.15 95% CI (0.05-0.49)]. (Table 1) WHO grading of the disease is shown in figure 1, there was a 100% mortality among patients on mechanical ventilator.

**Figure 1:**
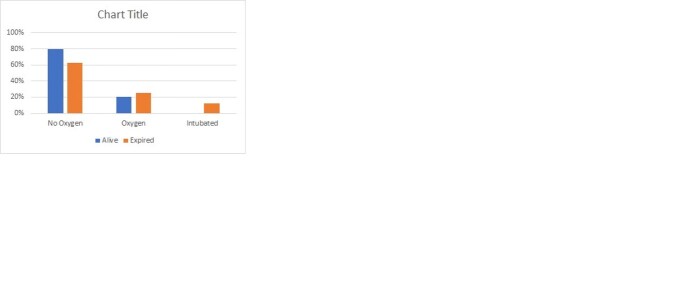
WHO clinical grading of severe disease

**Table 2:**
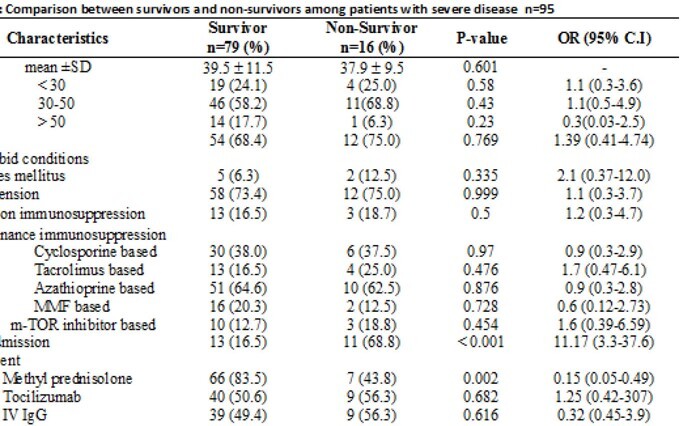
Comparison between survivors and non-survivors among patients with severe disease

**Conclusion:**

ICU admission and high D-dimers at the time of admission are the significant risk factors for mortality in patients with Covid-19 infection. There was no association of graft dysfunction with mortality. Steroids use has significantly improved survival in renal transplant recipients with severe Covid-19 infection.

**Disclosures:**

**All Authors**: No reported disclosures.

